# Inhibition of Human Cathepsins B and L by Caffeic Acid and Its Derivatives

**DOI:** 10.3390/biom11010031

**Published:** 2020-12-29

**Authors:** Liza Ulčakar, Marko Novinec

**Affiliations:** Department of Chemistry and Biochemistry, Faculty of Chemistry and Chemical Technology, University of Ljubljana, Večna pot 113, 1000 Ljubljana, Slovenia; liza.ulcakar@gmail.com

**Keywords:** enzyme inhibition, proteolysis, peptidase, catalytic inhibition, mixed inhibition, chlorogenic acid, caffeic acid phenethyl ester

## Abstract

Caffeic acid (CA) and its derivatives caffeic acid phenethyl ester (CAPE) and chlorogenic acid (CGA) are phenolic compounds of plant origin with a wide range of biological activities. Here, we identify and characterize their inhibitory properties against human cathepsins B and L, potent, ubiquitously expressed cysteine peptidases involved in protein turnover and homeostasis, as well as pathological conditions, such as cancer. We show that CAPE and CGA inhibit both peptidases, while CA shows a preference for cathepsin B, resulting in the strongest inhibition among these combinations. All compounds are linear (complete) inhibitors acting via mixed or catalytic mechanisms. Cathepsin B is more strongly inhibited at pH 7.4 than at 5.5, and CA inhibits its endopeptidase activity preferentially over its peptidyl-dipeptidase activity. Altogether, the results identify the CA scaffold as a promising candidate for the development of cathepsin B inhibitors, specifically targeting its endopeptidase activity associated with pathological proteolysis of extracellular substrates.

## 1. Introduction

Plants are a rich source of biologically active phenolic compounds. Many of these are known not only for their antioxidant properties [1] but also for their beneficial health effects in traditional medicine and as food supplements, as well as potential application in standard medicine. An important group of such compounds is cinnamic acid and its derivatives, which have antimicrobial, antitumor, anti-inflammatory and other biological activities [2,3,4,5]. At the molecular level, this is due to their well-known antioxidative properties [1,6] but also specific interactions with protein targets. For example, caffeic acid (CA; 3,4-dihydroxycinnamic acid) and its phenethyl ester (CAPE) exert their antitumor activities by inhibiting nuclear factor kappa B [7,8] and matrix metalloproteases (MMPs) -2 and -9 [9]. Both also inhibit other enzymes, including lipoxygenases [10,11,12], cyclooxygenases [13], xanthine oxidase [14], glutathione S-transferase [15], and tyrosine phosphatases [16].

Adding to this list, we have recently characterized CA and chlorogenic acid (CGA, an ester of CA and quinic acid) as inhibitors of dipeptidyl-peptidase I (DPPI) [17]. This enzyme is a member of the papain-like family of cysteine peptidases, which comprises eleven homologs in humans. In view of this, we expanded our investigation to cathepsins B and L, which, like DPPI, are ubiquitously and abundantly expressed in humans. Cathepsin B is unique within the family for being an endopeptidase or a peptidyl-dipeptidase, depending on the position of a structural insertion called the occluding loop, which changes conformation in a pH-dependent manner. At low pH (below 6), the occluding loop is bound into the active site and the enzyme functions as a peptidyl-dipeptidase. Above pH 6, the loop moves away from the active site and the enzyme functions as an endopeptidase [18]. The physiological functions of cathepsin B include non-specific protein turnover, antigen presentation, and thyroid hormone processing (reviewed in [19]). It was also shown to trigger apoptosis upon its release into the cytosol [20]. Cathepsin B is well known and has also been investigated for its involvement in pathological conditions, including its multiple roles in cancer [21], arthritis [22] and Alzheimer’s disease [23]. At the cellular level, altered trafficking of the peptidase is observed in both arthritis and cancer, leading to its secretion from the cell, where it can associate with the cell surface in caveolae and degrade various components of the extracellular matrix [24]. Cathepsin L is a predominantly lysosomal enzyme that plays a role in both non-specific protein turnover and specific proteolytic processing, e.g., activation of heparanase [25]. It is associated with numerous physiological and pathological processes, including adipogenesis and glucose tolerance, as well as obesity, diabetes, and cancer [26,27]. Together with cathepsin B, it facilitates the entry of certain viruses into the cell [28,29] and has recently attracted much attention for its role in the infection of cells by SARS coronavirus 2 [30]. Cathepsin L can also be secreted into the extracellular space from certain cells, e.g., macrophages and tumor cells. It exhibits elastinolytic activity [31,32], low collagenolytic activity [33], and proteolytic activity against other structural proteins of the extracellular matrix such as laminin and fibronectin [34].

In this work, we evaluated a series of cinnamic acids, including CA and CGA, as potential inhibitors of human cathepsins B and L. We demonstrated that these compounds inhibited both peptidases, but with very different affinities, and characterized the kinetic mechanisms of inhibition. Based on our results, we determined the structure–function relationship for the inhibition of both peptidases by these compounds.

## 2. Materials and Methods

### 2.1. Materials

All fluorogenic substrates were from Bachem (Bubendorf, Switzerland). Dithiothreitol (DTT) was from Merck (Darmstadt, Germany). The detailed list of tested compounds and their commercial sources is provided as Supplementary Table S1. Recombinant human cathepsins B and L were produced in-house according to procedures described in the following references [33,35].

### 2.2. Kinetic Measurements

All kinetic measurements were performed fluorimetrically using a PerkinElmer LS50B Fluorimeter (PerkinElmer Inc., Waltham, MA, USA). Assays were performed at 25 ± 1 °C in 1 × 1 cm^2^ single-use acrylic cuvettes with magnetic stirring using two different buffers: 50 mM Na-acetate pH 5.5, 1 mM EDTA, 2.5 mM DTT and 20 mM Na-phosphate buffer pH 7.4, 1 mM EDTA, and 2.5 mM DTT. The substrates used were Z-Phe-Arg-AMC for cathepsin B and Z-Leu-Arg-AMC for cathepsin L. We avoided Z-Phe-Arg-AMC for cathepsin L due to its minimal *K*_m_ value (below 1 µM), which makes it difficult to perform measurements at substrate concentrations at or below this value. AMC’s release was followed by an excitation wavelength λ_ex_ = 370 nm and an emission wavelength λ_em_ = 455 nm.

In addition, the peptidyl-dipeptidase activity of cathepsin B was measured with the internally quenched fluorigenic substrate Abz-Gly-Ile-Val-Arg-Ala-Lys(Dnp)-OH. Cleavage of this substrate was assessed at λ_ex_ = 370 nm and λ_em_ = 430 nm. The former deviated from the usual 320 nm due to strong absorption of most compounds at 320 nm, which was significantly lower at 370 nm. Nevertheless, appropriate correction of the inner filter effect was performed for all compounds according to the following reference [36].

The *K*_m_ values for the enzymes/substrate pairs used in this work were 2 µM for cathepsin L/Z-Leu-Arg-AMC, 23 µM for cathepsin B/Z-Phe-Arg-AMC at pH 5.5, 33 µM for cathepsin B/Z-Phe-Arg-AMC at pH 7.4, and 4 µM for cathepsin B/Abz-Gly-Ile-Val-Arg-Ala-Lys(Dnp)-OH, respectively.

### 2.3. Kinetic Data Analyses

All kinetic analyses were performed with GraphPad Prism 8 software (GraphPad Software, San Diego, CA, USA). The logistic equation adapted to enzyme activity [37] was used to determine the IC_50_ values of the tested compounds (X)

(1) $$v_{X}=v_{0}-\frac{\left(v_{0}-v_{\infty }\right)\times \left[X\right]}{{\mathrm{IC}}_{50}+\left[X\right]}$$

where *v*_X_ and *v*_0_ are reaction rates in the presence and absence of inhibitor X and *v*_∞_ is the reaction rate at saturation with inhibitor. For linear (complete) inhibitors, *v*_∞_ = 0.

The kinetic models used for determination of kinetic mechanisms were the models for linear catalytic (uncompetitive) and linear mixed inhibition, as defined by Equations (2) and (3), respectively:

(2) $$v_{X}=\frac{v_{0}\times \left(1+\sigma \right)}{1+\sigma \times \left(1+\frac{\left[X\right]}{K_{\mathrm{Ca}}}\right)}$$

(3) $$v_{X}=\frac{v_{0}\times \left(1+\sigma \right)}{1+\frac{\left[X\right]}{K_{\mathrm{Sp}}}+\sigma \times \left(1+\frac{\left[X\right]}{K_{\mathrm{Ca}}}\right)}$$

where *K*_Sp_ and *K*_Ca_ are inhibition constants for the specific (competitive) and catalytic (uncompetitive) components of the inhibitor, respectively, and *σ* = [S]/*K*_m_.

For the qualitative diagnosis of kinetic mechanisms, we used the specific velocity plot [38] defined by Equation (4)

(4) $$\frac{v_{0}}{v_{X}}=\frac{\left[X\right]\times \left(\frac{1}{\alpha \times K_{X}}-\frac{1}{K_{X}}\right)}{1+\beta \times \frac{\left[X\right]}{\alpha \times K_{X}}}\frac{\sigma }{1+\sigma }+\frac{1+\frac{\left[X\right]}{K_{X}}}{1+\beta \times \frac{\left[X\right]}{\alpha \times K_{X}}}$$

where the ratio *v*_0_/*v*_X_ represents specific velocity, *K*_X_ is the equilibrium dissociation constant of the EX complex, and *α* and *β* are dimensionless coefficients that define the effect of the modifier on the substrate binding affinity and catalytic rate of the enzyme, respectively. For linear inhibition mechanisms, *β* = 0. In relation to Equations (2) and (3), *K*_X_ = *K*_Sp_ and *α × K*_X_ = *K*_Ca_.

## 3. Results

### 3.1. Evaluation of Cinnamic Acid Derivatives as Cathepsin Inhibitors

**Figure 1:**
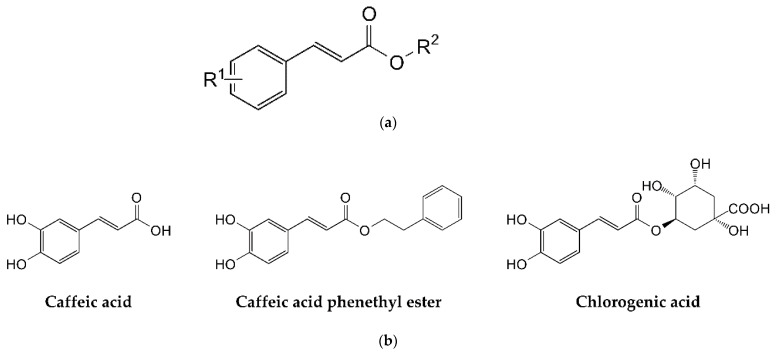
(**a**) The cinnamic acid scaffold; (**b**) chemical structures of cinnamic acid derivatives that acted as inhibitors of cathepsins B and/or L.

CA and CGA were previously characterized as linear, mixed balanced, and linear catalytic inhibitors of DPPI, respectively [17]. Herein, a series of compounds based on the cinnamic acid scaffold (Figure 1a), including CA and CGA, were tested for their inhibitory activity against human cathepsins B and L. An initial screening was performed at pH 5.5, which is optimal for the activity and stability of papain-like cysteine peptidases. We first determined that all active compounds acted as linear inhibitors and then determined their IC_50_ values at [S] = *K*_m_ in the case of cathepsin B and [S] = 2 × *K*_m_ in the case of cathepsin L. For linear inhibition mechanisms, the IC_50_ values at these substrate concentrations range from 1 × *K*_X_ to 3 × *K*_X_, where *K*_X_ is the appropriate inhibition constant (either *K*_Sp_ or *K*_Ca_; see Section 2.3 for details). The IC_50_ value is therefore a reasonable estimate of the true inhibition constant(s). All determined IC_50_ values are listed in Table 1, and the structures of the best compounds are shown in Figure 1b.

Among the compounds, CA stood out for its inhibitory effect against cathepsin B and the lack of inhibition of cathepsin L, while CAPE and CGA weakly inhibited both enzymes. These compounds were selected for further characterization. In addition to these results, 2,4-dihydroxycinnamic acid showed some activity against both peptidases. However, since its effect was significantly weaker, it was not further characterized. The remaining compounds had very weak or no effect on enzyme activity, with estimated IC_50_ values of more than 1 mM. Despite the limited number of tested compounds, this selection was sufficient to identify the basic structural features that contribute to the inhibition of cathepsins B and L. The comparison of structures and IC_50_ values shows that the presence of two hydroxyl groups in the m- and p-positions at site R^1^ is optimal for efficient inhibition of cathepsin B. Removal of these groups or their replacement by methoxy groups abolished the affinity for cathepsin B. The presence of bulky groups (phenylethyl group or quinic acid) at site R^2^ reduced the affinity for cathepsin B, while reduction of the alkene group abolished it completely. In contrast, inhibition of cathepsin L required the presence of additional functional groups at site R^2^. Thus, site R^2^ determines the selectivity of the compounds for individual papain-like peptidases.

### 3.2. Kinetic Characterization of Selected Compounds

**Figure 2:**
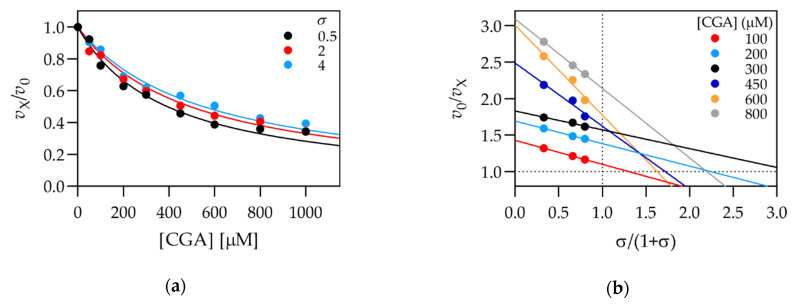
Kinetic characterization of the inhibition of cathepsin L by chlorogenic acid (CGA). (**a**) Titration curves produced at three different substrate concentrations. Decreasing inhibitor efficiency with increasing substrate concentration indicates predominantly specific inhibition; (**b**) replot of the data in panel (**a**) in the form of the specific velocity plot. Straight lines (corresponding to individual inhibitor concentrations) intersect the *y* = 1 axis at *x* values larger than 1, indicating a mixed, predominantly specific mechanism. *σ* equals [S]/*K*_m_. Experiments were performed at 25 ℃, in 50 mM Na-acetate buffer pH 5.5, with 1 mM EDTA and 2.5 mM DTT.

The kinetic characterization of CA, CAPE, and CGA was initially performed under the same conditions as the inhibition screening (pH 5.5). All compounds acted as fast, linear inhibitors of cathepsin L, as observed for DPPI [17]. Kinetic mechanisms and inhibition parameters were determined by a combination of titration curves (plots of the residual enzyme activity (*v*_X_/*v*_0_) against the inhibitor concentration) and replots of the experimental data as specific velocity plots [38]. The analysis of CGA is shown as an example in Figure 2, and all calculated kinetic parameters are summarized in Table 2. The remaining titration curves were collected in Supplementary Figure S1. All compounds acted as linear, mixed inhibitors with a dominant specific component (*K*_Sp_ < *K*_Ca_). All calculated inhibition constants were in the range of 10^−4^ M and were therefore comparable to those determined for inhibition of DPPI [17]. In accordance with the IC_50_ values in Table 1, CAPE was the strongest inhibitor, with a *K*_Sp_ value of 120 ± 70 µM. However, a more precise characterization of its effect was made difficult by its limited solubility of only up to about 200 µM under the experimental conditions used.

**Figure 3:**
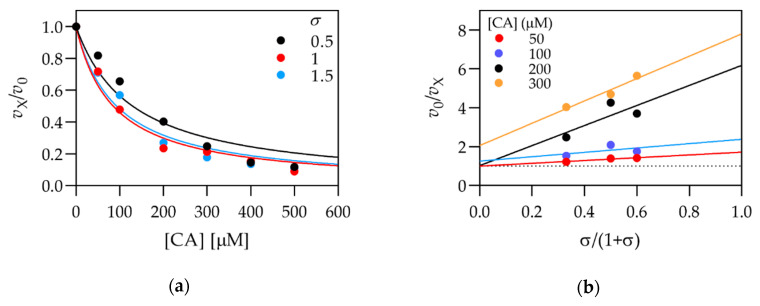
Kinetic characterization of the inhibition of cathepsin B by CA at pH 7.4: (**a**) Titration curves produced at three different substrate concentrations. Increasing inhibitor efficiency with increasing substrate concentration indicates predominantly catalytic inhibition; (**b**) replot of the data in panel (**a**) in the form of the specific velocity plot. Straight lines (corresponding to individual inhibitor concentrations) intersect the *y* = 1 axis at *x* values near 0, indicating a pure catalytic mechanism. *σ* equals [S]/*K*_m_. Experiments were performed at 25 ℃ in 20 mM Na-phosphate buffer pH 7.4 with 1 mM EDTA and 2.5 mM DTT.

Cathepsin B was inhibited by CAPE and CGA with similar affinities as cathepsin L (Table 2), except that CAPE acted as a catalytic inhibitor. In contrast to cathepsin L, CA was the most potent inhibitor of cathepsin B and acted as a mixed inhibitor with a dominant catalytic component (*K*_Ca_ < *K*_Sp_). To account for the reversible pH-dependent binding of the occluding loop to the active site, we performed the kinetic characterization of the inhibition of cathepsin B by the above-mentioned compounds at pH 7.4. The characterization of CA is shown in Figure 3, and the other results are collected in Supplementary Figure S1. The calculated kinetic parameters (Table 2) show that all compounds inhibited cathepsin B more strongly at pH 7.4 than at pH 5.5. All inhibition constants were in the 10^−5^ M range, with CA retaining the highest affinity for cathepsin B. For cathepsin L, no such trend was observed (results not shown), indicating that the pH-dependent increase in affinity is specific for cathepsin B. The inhibition by CA was satisfactorily described by pure catalytic inhibition, and CAPE and CGA acted as mixed inhibitors. We also determined the inhibition of the peptidyl-peptidase activity of cathepsin B using the substrate Abz-Gly-Ile-Val-Arg-Ala-Lys(Dnp)-OH at pH 5.5 and [S] = *K*_m_. The determined IC_50_ value for CA was > 1 mM, indicating that CA specifically inhibits the endoproteolytic activity of cathepsin B. Conversely, IC_50_ values for CAPE and CGA were 100 ± 20 µM and 360 ± 60 µM, respectively, which is comparable to the values obtained with Z-Phe-Arg-AMC (see Table 1). CAPE and CGA thus indiscriminately inhibit both the endoproteolytic and exoproteolytic activity of cathepsin B.

## 4. Discussion

Caffeic acid and its derivatives have numerous biological activities, including antioxidant, antitumor, and anti-inflammatory. Accordingly, these compounds can act by modulating multiple macromolecular targets, including peptidases. CA and CAPE were identified as inhibitors of gelatinases (MMPs-2 and -9), with IC_50_ values in the micromolar range and good antitumor activity in vitro and in vivo in mice [9]. Weaker inhibition of other MMPs and no inhibition of cathepsin K were observed. The latter observation was also confirmed in our laboratory in the context of this work (results not shown). However, we have recently observed weak inhibition of DPPI (also known as cathepsin C) by CA and CGA [17]. Herein, we extended our analysis to cathepsins B and L. CGA and CAPE inhibited both enzymes, while CA showed a strong preference for cathepsin B. According to our analysis, the presence of an additional group at site R^2^ (the alkyl group of the CA ester; see Figure 1) is necessary for efficient inhibition of cathepsin L but negatively affects the affinity for cathepsin B, which explains the selectivity of CA. Kinetically, all compounds were mixed or catalytic inhibitors. This seems to be their preferred mechanism of action not only against peptidases [17] but also against other enzymes. For example, CA was a mixed balanced (non-competitive) inhibitor of 5-lipoxygenase [10], while CAPE was a catalytic (uncompetitive) inhibitor for the same enzyme [11]. In the case of xanthine oxidase, CGA was a mixed inhibitor, but CA and CAPE were specific (competitive) inhibitors [39]. When considering the potential use of these compounds in vivo, it should be taken into account that endogenous esterases hydrolyse both CAPE and CGA to CA and other by-products [40,41]; therefore, CA is the primary biologically active substance in vivo.

The most promising result of this work is that the inhibition of cathepsin B by CA is not only selective over other ubiquitously expressed cysteine cathepsins but CA also preferentially inhibits the endoproteolytic activity of cathepsin B without affecting the peptidyl-dipeptidase activity. Like MMPs -2 and -9, cathepsin B is associated with increased extracellular proteolysis in cancer and arthritis [21,22] and has been considered a potential target for their treatment for decades. Apart from specific inhibitors that occupy the active site in a substrate-like manner, strategies have emerged in recent years that target other sites on cathepsin B. Schenker and colleagues discovered the compound DOFA, which targets the occluding loop to stabilize its binding to the active site, thereby restricting the enzyme to exopeptidase activity [42]. In addition, nitroxoline and a series of its derivatives that bind to the S’ sites were described and showed a variety of mixed and uncompetitive effects [43,44,45]. Recently, a designed ankyrin repeat protein was constructed that binds outside the active centre and acts as a partially mixed inhibitor [46]. Of these, CA’s effect is most similar to nitroxoline and we assume that it may also bind to the S’ sites. Such a binding mode would be supported by its kinetic mechanism of action. However, this assumption requires experimental confirmation before further conclusions and comparisons can be drawn. The effect that distinguishes CA from nitroxoline is the specific inhibition of the endoproteolytic activity of cathepsin B, which is not observed with the latter. Altogether, the results of this work expand the versatility of the CA scaffold [47] to the synthesis of specific cathepsin B inhibitors targeting only the endopeptidase activity associated with the pathological proteolysis of extracellular substrates.

## Figures and Tables

**Table 1:** Determined IC_50_ values of all tested compounds: The activity of cathepsin B was measured using the substrate Z-Phe-Arg-AMC at [S] = *K*_m,_ and the activity of cathepsin L was measured using the substrate Z-Leu-Arg-AMC at [S] = 2 × *K*_m_. All experiments were performed at 25 °C in 50 mM Na-acetate buffer pH 5.5 with 1 mM EDTA and 2.5 mM DTT.

Common Name	R ^1^	R ^2^	Cathepsin B IC_50_ (µM)	Cathepsin L IC_50_ (µM)
Cinnamic acid	None	H-	>1000	>1000
*o*-Coumaric acid	2-hydroxy-	H-	>1000	>1000
*m*-Coumaric acid	3-hydroxy-	H-	>1000	>1000
*p*-Coumaric acid	4-hydroxy-	H-	>1000	>1000
2,4-Dihydroxy- cinnamic acid	2,4-dihydroxy-	H-	910 ± 30	930 ± 150
Caffeic acid ^2^	3,4-dihydroxy-	H-	110 ± 10	>1000
Hydrocaffeic acid ^1^	3,4-dihydroxy-	H-	>1000	>1000
Ferulic acid	3-methoxy-, 4-hydroxy-	H-	>1000	>1000
Sinapinic acid	3,3′-dimethoxy-, 4-hydroxy-	H-	>1000	>1000
Caffeic acid phenethyl ester ^2^	3,4-dihydroxy-	2-phenylethyl-	470 ± 30	190 ± 30
Chlorogenic acid ^2^	3,4-dihydroxy-	3-*O*-quinic acid	340 ± 20	480 ± 20

^1^ Contains reduced alkene group (IUPAC name 3-(3,4-Dihydroxyphenyl) propionic acid). ^2^ Chemical structures shown in Figure 1b.

**Table 2:** Kinetic parameters for the inhibition of cathepsins B and L by caffeic acid (CA), caffeic acid phenethyl ester (CAPE), and chlorogenic acid (CGA). Parameters were determined by non-linear regression analysis of titration curves recorded at three different substrate concentrations using Equations (2) and (3).

Enzyme/Inhibitor	pH	Mechanism	*K*_Sp_ (µM)	*K*_Ca_ (µM)
Cathepsin L				
CA	5.5	Mixed	740 ± 70	1270 ± 120
CAPE	5.5	Mixed	120 ± 70	170 ± 30
CGA	5.5	Mixed	330 ± 20	670 ± 60
Cathepsin B				
CA	5.5	Mixed	270 ± 140	82 ± 16
	7.4	Catalytic	n.a.^1^	46 ± 4
CAPE	5.5	Catalytic	n.a.^1^	140 ± 10
	7.4	Mixed	107 ± 38	70 ± 19
CGA	5.5	Mixed	340 ± 80	1200 ± 1100
	7.4	Mixed balanced	92 ± 15	92 ± 15

^1^ n.a.—not applicable, i.e., *K*_Sp_ approaches ∞ for a pure linear catalytic mechanism.

## Data Availability

Data is contained within the article or supplementary material.

## Supplementary Materials

Supplementary materials can be found at https://www.mdpi.com/2218-273X/11/1/31/s1. Supplementary Materials, Figure S1: Titrations of cathepsins B and L with caffeic acid (CA), caffeic acid phenethyl ester (CAPE) and chlorogenic acid (CGA); Table S1: List of tested compounds and their commercial sources.

## Abbreviations

Abz	aminobenzoyl
AMC	7-amino-4-methylcoumarin
CA	caffeic acid
CAPE	caffeic acid phenethyl ester
CGA	chlorogenic acid
Dnp	2,4-dinitrophenylamino
DPPI	dipeptidyl-peptidase I
DTT	dithiothreitol
Z	benzyloxycarbonyl
