# Supplementary material for: Inhibition of Human Cathepsins B and L by Caffeic Acid and Its Derivatives
Source: Biomolecules. 2020 Dec 29;11(1):31. doi: 10.3390/biom11010031 (PMC7824550; doi:10.3390/biom11010031)
Supplement: Supplementary file 1 [file biomolecules-11-00031-s001.pdf]

Inhibition of human cathepsins B and L by caffeic acid and its derivatives

Liza Ulčakar, Marko Novinec

University of Ljubljana, Faculty of Chemistry and Chemical Technology, Department of Chemistry and Biochemistry, Večna pot 113, 1000 Ljubljana, Slovenia

---

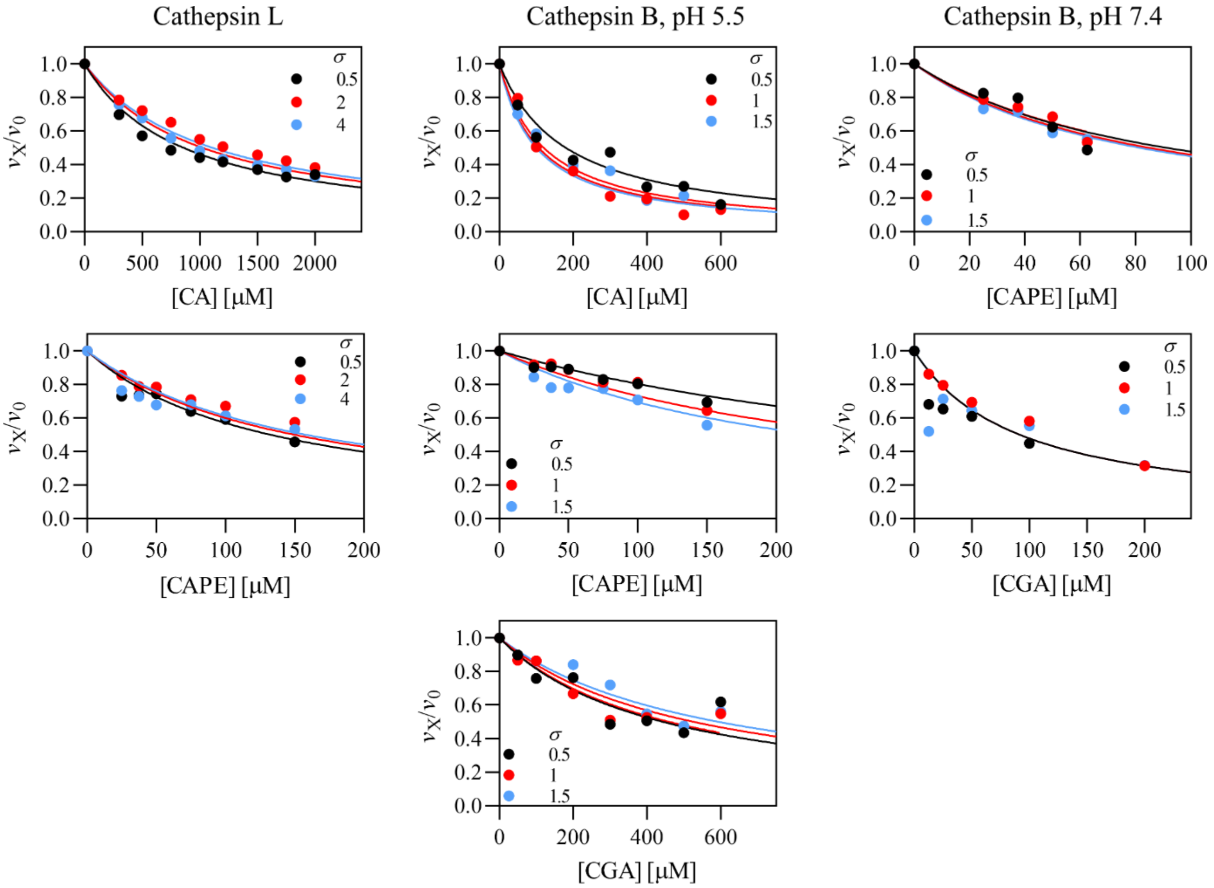

<u>Supplementary Figure S1:</u> Titrations of cathepsins B and L with caffeic acid (CA), caffeic acid phenethyl ester (CAPE) and chlorogenic acid (CGA). Titration curves were recorded at three different substrate concentrations. σ equals $[S]/K_m$. Both cathepsins were assayed in 50 mM Na-acetate **pH 5.5** containing 1 mM EDTA and 2.5 mM DTT. Additionally, cathepsin B was assayed in 20 mM Na-phosphate buffer **pH 7.4** containing 1 mM EDTA and 2.5 mM DTT. The substrates used were Z-Phe-Arg-AMC for cathepsin B and Z-Leu-Arg-AMC for cathepsin L, respectively. All assays were performed at 25 ± 1 °C.

<u>Supplementary Table S1:</u> List of tested compounds and their commercial sources.

**Compound**	**Source**	**Product no.**
Cinnamic acid	Merck (Germany)	C80857
o-Coumaric acid	Merck (Germany)	H22809
m-Coumaric acid	Merck (Germany)	H23007
p-Coumaric acid	Merck (Germany)	C9008
2,4,-Dihydroxycinnamic acid	Merck (Germany)	663158
Caffeic acid[2]	Merck (Germany)	C0625
Hydrocaffeic acid[1]	Merck (Germany)	102601
Ferulic acid	Merck (Germany)	128708
Sinapinic acid	Merck (Germany)	D7927
Caffeic acid phenethyl ester[2]	Carl Roth (Germany)	5465
Chlorogenic acid[2]	Merck (Germany)	C3878
